# Differences in Fecal Short-Chain Fatty Acids between Alcoholic Fatty Liver-Induced Cirrhosis and Non-alcoholic (Metabolic-Associated) Fatty Liver-Induced Cirrhosis

**DOI:** 10.3390/metabo13070859

**Published:** 2023-07-19

**Authors:** Xinlu Cao, Oksana Zolnikova, Roman Maslennikov, Maria Reshetova, Elena Poluektova, Arina Bogacheva, Maria Zharkova, Vladimir Ivashkin

**Affiliations:** 1 Department of Internal Medicine, Gastroenterology and Hepatology, Sechenov University, 119435 Moscow, Russia; tsao_s@student.sechenov.ru (X.C.); zolnikova_o_yu@staff.sechenov.ru (O.Z.); reshetova_m_s@staff.sechenov.ru (M.R.); poluektova_e_a@staff.sechenov.ru (E.P.); bogacheva_a_v@staff.sechenov.ru (A.B.); zharkova_m_s@staff.sechenov.ru (M.Z.); ivashkin_v_t@staff.sechenov.ru (V.I.); 2 The Interregional Public Organization “Scientific Community for the Promotion of the Clinical Study of the Human Microbiome”, 119121 Moscow, Russia

**Keywords:** liver cirrhosis, short-chain fatty acids, gut microbiota, gut–liver axis

## Abstract

The objective of this study was to investigate the metabolic activity of the gut microbiota in cirrhosis due to different variants of fatty liver disease (alcoholic vs. non-alcoholic [metabolic-associated] one [AFLD and MAFLD]). The present study included 24 patients with alcoholic liver cirrhosis, 16 patients with MAFLD-related cirrhosis, and 20 healthy controls. The level and spectrum of short-chain fatty acids (SCFAs) were determined via gas–liquid chromatography. All patients with cirrhosis showed a decrease in the total content of SCFAs (*p* < 0.001) and absolute content of acetate (*p* < 0.001), propionate (*p* < 0.001), butyrate (*p* < 0.001), and isovalerate (*p* < 0.001). In MAFLD cirrhosis, the metabolic activity of the microbiota was significantly altered compared to patients with alcoholic cirrhosis, as evidenced by a lower total SCFA content (*p* < 0.001) and absolute content of acetate (*p* < 0.001), propionate (*p* < 0.001), and butyrate (*p* < 0.001); a higher relative content of isovalerate (*p* < 0.001); and a higher IsoCn/Cn ratio (*p* < 0.001). Various clinical and laboratory parameters correlate differently with fecal SCFAs and their fractions in cirrhosis due to AFLD and MAFLD. SCFA-producing metabolic activity is reduced more in MAFLD cirrhosis than in alcoholic cirrhosis. According to the etiological factors of cirrhosis, disorders of this metabolic activity may be involved in different pathogenetic pathways.

## 1. Introduction

In recent decades, chronic liver disease has emerged as a significant global health concern [1,2]. Increasing evidence suggests that interactions between the intestinal microbiota, its metabolites, the immune system, and the liver play a pivotal role in the pathogenesis of both alcoholic liver disease (ALD) and metabolic-associated fatty liver disease (MAFLD) [2,3,4,5,6,7,8]. The bidirectional homeostasis of the gut–liver axis reflects the intimate relationship between the gut and the liver. In the gut–liver axis, complex interactions exist between the gut microbiota, immune system, and muco-epithelial barrier [9,10,11,12,13,14,15,16].

As cirrhosis progresses, the composition of the bacterial microbiota varies, including a decrease in the number of commensal bacteria and an increase in the number of conditionally pathogenic and pathogenic microorganisms, also known as pathobionts. The disruption of the intestinal barrier due to increased intestinal permeability is a factor that facilitates the portal influx of pathogen-associated molecular patterns (PAMPs), thereby causing the entry of bacterial endotoxins, lipopolysaccharides, and bacterial metabolites into the liver and eliciting a proinflammatory immune response that exacerbates hepatocyte damage.

Several studies have demonstrated a correlation between microbiota disruption and the progression of liver cirrhosis. In patients with cirrhosis of the liver, the diversity of the intestinal biotope and a decrease in the presence of commensal taxa belonging to the *Firmicutes* and *Bacteroidetes* categories are diminished [17,18], and this is accompanied by the proliferation of pathogenic microorganisms such as *Enterobacteriaceae, Veillonellaceae*, and *Streptococcaceae* [19]. In other studies [6,7,8,10,12,15], similar results have been obtained regarding microbiota diversification. In addition, the literature suggests that the decompensation of chronic liver disease correlates with changes in the bacterial composition of the microbiota, thereby deteriorating the prognosis of the disease [7,17,20].

Changes in the composition of the microbiota are associated with disturbances in its metabolic activity, particularly in the synthesis of short-chain fatty acids (SCFAs), the primary bacterial metabolites. During the fermentation of dietary fiber by intestinal bacteria, SCFAs (acetate, propionate, butyrate) are produced. The majority (90–95%) of SCFAs are absorbed in the colon, where they not only provide energy to the intestinal epithelium but also perform a variety of biological functions. For example, SCFAs regulate the homeostasis of the gut–liver axis by maintaining intestinal permeability and regulating immunity, lipogenesis, and gluconeogenesis [3,5,9,10]. It has been hypothesized that, even in the presence of cirrhosis and hepatitis, SCFAs may modulate liver injury [8].

In this study, we analyzed and compared the concentrations of SCFAs in the feces of patients with alcoholic liver cirrhosis and MAFLD-related cirrhosis to those of healthy controls.

## 2. Materials and Methods

The study was conducted in accordance with the Declaration of Helsinki and was approved by the ethics committee of Sechenov University (No. 01-22 of 24 January 2022). All participants signed an informed consent form. 

Regarding inclusion criteria, all patients who were admitted to the Hepatology Department of Sechenov University from 10 December 2022 to 25 March 2023 with a diagnosis of cirrhosis were screened. The diagnosis of cirrhosis was established on the basis of liver biopsy data or a combination of clinical, laboratory, and instrumental data.

The criteria for inclusion were age 18–75 years and class A–B cirrhosis (according to the Child–Pugh score) due to AFLD or MAFLD. There are no published data that could be used to calculate the required number of patients.

To exclude a different etiology of cirrhosis, all patients were tested for the presence of markers of viral hepatitis, autoimmune hepatitis, hemochromatosis (transferrin saturation with iron), and Wilson’s disease (indicators of copper metabolism). The diagnosis of alcoholic fatty liver disease was established by detecting alcohol consumption in hepatotoxic doses for >6 months (>30 g/day for men and >20 g/day for women [21]) and fatty liver according to elastometry data. The diagnosis of non-alcoholic fatty liver disease was established by detecting fatty liver according to elastometry data, with the denial of alcohol consumption in hepatotoxic doses and the presence of signs of metabolic disorders (hyperglycemia and/or hyperlipidemia). Patients with a possible overlap (alcoholic + non-alcoholic fatty liver disease), namely the use of alcohol in hepatotoxic doses and the presence of diabetes, were excluded from the study.

The study excluded patients who took drugs that affect the composition of the gut microbiota (probiotics, prebiotics, antibiotics, prokinetics and PPIs) within the last 3 months leading up to the start of the study, as well as those with concomitant diseases in which there is a change in the composition and metabolic function of the intestinal microbiota (except for diabetes mellitus, hypertension, and hyperuricemia in patients with MAFLD).

Of the total 100 patients, 60 did not meet the criteria: cirrhosis of viral (n = 23), autoimmune (n = 9), and mixed (n = 6) etiology, cancer (n = 7), use of drugs that affect the composition of the gut microbiota (n = 15).

The control group (the CON group) consisted of 20 healthy volunteers with no gastrointestinal tract issues; no concomitant diseases of the respiratory, urinary, endocrine, cardiovascular system; and no oncological diseases. All volunteers applied to the clinic for a preventive examination.

In all study participants, the absolute and relative content of acetic (C2), propionic (C3), and butyric (C4) acids; the level of isoacids (SCFA isomers); and the ratio of isoacids to unbranched acids (isoCn/Cn) were determined using the following method: Fecal samples were collected from all study participants and kept at −80 °C until further analysis. After defrosting, a 0.1 g fecal sample was placed in a tube with a conical bottom; 2 mL distilled water and 1 mL calibration solution were added before it was mixed by shaking for 10 min. Further, 0.5 mL of 1 N HCl was added, and the mixture was then centrifuged at 5000 rpm for 10 min. Next, 1 μL supernatant was injected with a microsyringe into the Khromos GCh-1000 gas chromatograph evaporator with a flame ionization detector equipped with a 36 meter-long quartz capillary column with an inner diameter of 0.32 mm with a stationary free fatty acid phase in the form films, which were 0.33 µm thick. The chromatograph operation mode was isothermal, with a thermostat temperature of 150 °C and an evaporator and detector temperature of 230 °C. The carrier gas was nitrogen, and the column inlet pressure was set to 1.8 atm. The carrier gas flow was 2.0 mL/min; the air flow was 300 mL/min. Chromatography took about 8 min. We determined the absolute content of individual acids in the mixture by calculating the areas of chromatographic peaks both by using the “triangle” method and by processing the chromatograms using a computer [22].

Patients also underwent standard laboratory and instrumental examinations.

Statistical data processing was carried out using the STATISTICA 10 software (StatSoft Inc., Tulsa, OK, USA) and GraphPad Prism 9 (GraphPad Software, Boston, MA, USA). Data are presented as median [interquartile range]. The significance of differences between the two groups was assessed by using the Mann–Whitney test. Differences in categorical variables were determined using Fisher’s exact test. Correlation analysis was performed using the Spearman method. If the probability of making a Type I error was *p* < 0.05, the differences were considered significant.

## 3. Results

The study included 24 patients with cirrhosis due to alcoholic fatty liver disease (ALC group), 16 patients with cirrhosis due to non-alcoholic (metabolic-associated) fatty liver disease (MET group), and 20 healthy controls (CON group). There was no significant difference between the patients and controls in age and sex distribution. Patients with cirrhosis had a higher BMI, a higher waist circumference, a higher level of triglycerides in the blood, higher ALT, higher AST, and higher glucose levels compared to the healthy individuals (Table 1).

Patients with MAFLD had higher values for body mass index, waist circumference, fibrinogen level, lymphocyte count, serum triglycerides, glucose, and IgG but lower serum levels of total bilirubin, uric acid, and IgM than patients with AFLD. There was no significant difference between these groups in other parameters of cirrhosis (Table 2).

The fecal levels of SCFAs, acetate, propionate, and butyrate were reduced in patients with cirrhosis compared to the healthy individuals, and this decrease was more pronounced in MAFLD than in alcohol cirrhosis. The fecal isoacid level was also lower in patients with cirrhosis compared to that of the healthy subjects, but there was no significant difference between patients with different etiologies of cirrhosis. The level of acetate did not differ significantly between the studied groups; the level of propionate in patients with cirrhosis was higher, and the level of butyrate was lower in cirrhosis patients than in the healthy individuals. At the same time, there was no significant difference between the patients with cirrhosis of various etiologies in the relative proportion of these SCFAs. The isoacid fraction was higher in patients with cirrhosis due to MAFLD than in patients with cirrhosis due to alcoholic liver disease and the healthy controls, and no significant difference was observed between the latter two groups of patients (Table 3).

**Figure 1:**
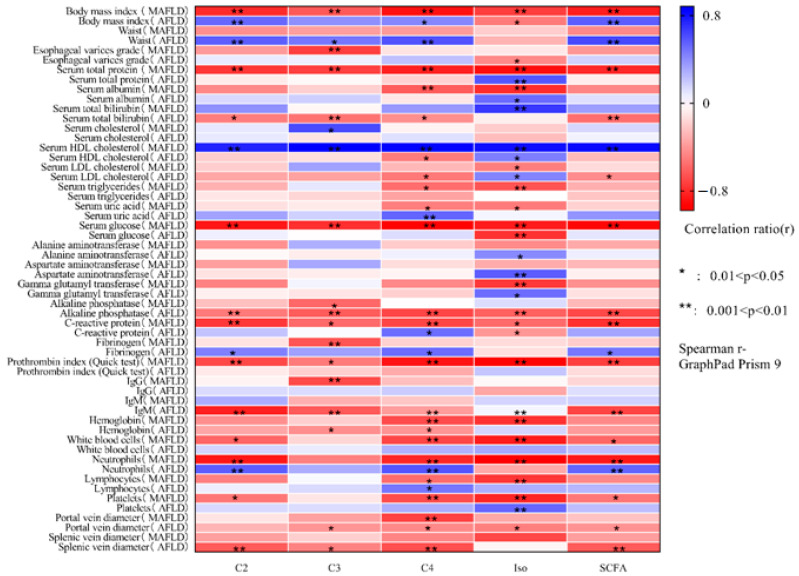
Correlation matrix of absolute fecal levels of acetate (C2), propionate (C3), butyrate (C4), isoacids (Iso), and all short-chain fatty acids (SCFAs) with the main indicators of cirrhosis due to alcoholic liver disease and non-alcoholic (metabolic-associated) fatty liver disease.

The correlation matrices of the fecal levels of SCFAs and their respective fractions with respect to the main indicators of cirrhosis in AFLD and MAFLD are presented in Figure 1.

Body mass index was directly correlated with the level of acetate (*p* = 0.008) and SCFA (*p* = 0.004) in AFLD and inversely correlated with the level of SCFA and all of its fractions in MAFLD (*p* < 0.01). Waist circumference was directly correlated with the level of SCFAs and almost all of their fractions in AFLD (*p* < 0.01), but its negative correlation with the level of SCFA in MAFLD could not reach the limit of significance.

Albumin levels were directly correlated with isoacid levels in AFLD patients and inversely correlated with that of the MAFLD group. The level of total bilirubin was inversely correlated with SCFAs and almost all of their fractions in AFLD and directly correlated with the level of isoacids in MAFLD.

The level of HDL cholesterol was positively correlated with the levels of SCFAs and all their fractions in MAFLD and only with the level of isoacids in AFLD. Additionally, the level of HDL cholesterol was inversely correlated with the level of butyrate in AFLD. The level of LDL cholesterol was inversely correlated with the levels of SCFAs and butyrate in AFLD, as well as isoacids in MAFLD, and was directly correlated with the level of isoacids in AFLD. Triglyceride levels were inversely correlated with butyrate and isoacid levels in MAFLD and did not correlate with any tested gut microbial metabolic index in AFLD. Uric acid levels were positively correlated with butyrate levels in AFLD and negatively correlated with that of MAFLD. The level of glucose was negatively correlated with the level of SCFAs and all their fractions in MAFLD and only with the level of isoacids in AFLD.

The level of alkaline phosphatase was negatively correlated with the level of SCFAs and all their fractions in AFLD and only with the level of propionate in MAFLD. The levels of ALT and AST were positively correlated with the levels of isoacids only in AFLD. The level of GGT was negatively correlated with the level of isoacids only in MAFLD.

The level of C-reactive protein was negatively correlated with the level of SCFAs and all their fractions in MAFLD and with the level of SCFA and isoacids in AFLD, but it was positively correlated with the level of butyrate in AFLD.

The prothrombin index was negatively correlated with the level of SCFAs and all their fractions only in MAFLD.

The diameter of the portal and splenic veins was inversely correlated with the level of SCFAs in almost all of their fractions in AFLD and with only one fraction of SCFAs in MAFLD.

Complete blood count parameters and serum IgG and IgM levels also correlated differently with the tested indicators of metabolic activity of the gut microbiota in AFLD and MAFLD.

There were no significant correlations between fecal SCFA levels (total and their fractions) and the tested parameters among the healthy controls.

Among patients with cirrhosis due to AFLD, fecal levels of SCFAs and their fractions were not associated with minimal hepatic encephalopathy, while among patients with cirrhosis due to MAFLD, the presence of minimal hepatic encephalopathy was associated with higher fecal levels of SCFAs, acetate, and isoacids (Table 4).

The presence of ascites was not associated with fecal levels of SCFAs and their fractions in cirrhosis due to either AFLD or MAFLD.

## 4. Discussion

In our study, we investigated the level of SCFAs in patients with Child–Pugh class A–B cirrhosis with different etiological factors. We examined patients with alcoholic cirrhosis and MAFLD-related cirrhosis. For both groups, the total content of SCFAs decreased (*p* < 0.001), as did the absolute content of acetate (*p* < 0.001), propionate (*p* < 0.001), butyrate (*p* < 0.001), and isoacids (*p* < 0.001).

In alignment with our results, M. Jin et al. previously reported a decrease in the content of SCFAs in the feces of patients with liver cirrhosis that correlated with disease severity [9]. Similar alterations were discovered in the blood serum of patients with liver cirrhosis, as SCFA levels were lower than in patients with MAFLD at the pre-cirrhotic stages [23].

To the best of our knowledge, no previous studies have investigated the level of SCFAs depending on the etiological factor of cirrhosis. In our study, we discovered that the metabolic activity of the microbiota changed substantially more in patients with MAFLD cirrhosis than in patients with alcohol-induced cirrhosis. In the MAFLD cirrhosis patients, the total content of SCFAs (*p* < 0.001) and the absolute content of acetate (*p* < 0.001), propionate (*p* < 0.001), and butyrate (*p* < 0.001) were lower than in the alcoholic cirrhosis patients. In the MAFLD cirrhosis patients, the relative content of isoacids (*p* < 0.001) and IsoCn/Cn (*p* < 0.001) was higher than in the alcoholic cirrhosis patients.

We gauged the correlations between the metabolic activity of the microbiota and routine clinical investigations, demonstrating that the gut microbiota may be involved in a variety of pathogenetic pathways depending on the etiological factor of cirrhosis.

It is known that, under normal conditions, the SCFAs acetate, propionate, and butyrate are formed in proportions of 60:20:18, respectively [24]. Changes in the relative content of these major SCFAs may indicate the predominance of the metabolic activity of aerobic or anaerobic flora [14]. According to the results of our study, the ratio of the SCFAs in both types of cirrhosis went unchanged, which was unexpected. After reading previously published publications, we did not discover any information regarding alterations in the profile of SCFAs in cirrhosis patients.

As for isoacids and the IsoCn/Cn ratio, they are normally formed in minimal quantities in the intestine. Their significant increase (*p* < 0.001) observed in patients with MAFLD-related cirrhosis indicates alterations in the intestinal mucosal epithelial layer. It is well known that epithelial mucus serves as a metabolic substrate for proteolytic microbiota [25]. Therefore, the changes observed indicate an increase in the activity of bacteria with pronounced proteolytic potential.

Currently, SCFAs are recognized as important molecules for maintaining intestinal barrier function [4,5,9]. A decrease in their content mediates an increase in intestinal permeability and leads to an increase in the portosystemic circulation of toxic and pro-inflammatory factors that contribute to liver damage and the development of complications in cirrhotic patients (bacterial translocation, hepatic encephalopathy). After the formation of SCFAs, they are absorbed by the epithelium of the colon and subsequently metabolized in the epithelium of the colon, liver, and peripheral muscle tissues [3,5]. SCFAs (mainly butyrate) are used as an energy source by the epithelial cells of the colon. Acetate, propionate, and butyrate residues are transported to the liver. Propionate serves as a substrate for gluconeogenesis and inhibits cholesterol synthesis, while acetate is used as a substrate for the synthesis of long-chain fatty acids, glutamine, glutamate, and beta-hydroxybutyrate [13,14,15]. Butyrate is directly oxidized by hepatocytes, thereby preventing high toxic systemic concentrations [14]. In one study, the interorgan exchange of SCFAs was measured in 22 patients (in vivo). According to the results of the study, the majority of butyrate remaining after absorption in the intestine and all of the propionate produced are effectively absorbed by the healthy liver parenchyma. Further analysis of splanchnic blood flow revealed statistically significant lower levels of butyrate and propionate in comparison to the portal system. However, the authors of this study noted that the level of acetate remained high even after passing through the liver [26].

The results of studies on the role of acetate in metabolism are somewhat contradictory. In addition to being a metabolite of the intestinal microbiota, acetate can also be a product of the liver’s metabolism of exogenous and endogenous ethanol. Apparently, this can lead to persistent concentrations of acetate in peripheral blood after enterohepatic circulation. Interestingly, in a study involving a healthy cohort of patients, an increase in the levels of ethylglucuronide and ethanol, as well as acetate, was observed when analyzing the metabolomic profile of urine in the context of consuming alcoholic beverages [27], whereas patients with cirrhosis experienced a decrease in acetate levels during our study. In many respects, this may be attributable to a decline in the liver’s detoxification capacity. Attempts have been made to evaluate the effects of acetate on peripheral tissue metabolism, inflammation, and hepatic lipid accumulation. In one study, the authors demonstrated that culturing a cell culture in a medium containing a high concentration of acetate led to the subsequent release of pro-inflammatory cytokines IL6, IL8, and TNF-α. Ethanol had similar effects [28]. The results of another experiment on an animal model indicate that acetate has a positive effect on lipogenesis in the liver, and against the background of acetate administration, the levels of aspartataminotransferase and alkaline phosphatase in the blood serum decreased [29]. To a certain extent, these data correspond to the results of our study, which can be summarized as follows: against the background of a decrease in the level of acetate, there was an increase in the level of alkaline phosphatase in the group of patients with alcoholic liver cirrhosis.

The limitations of our study include the small number of participants and a limited sample with respect to disease stage (Child–Pugh classes A–B). Further studies are needed to clarify the mechanisms of the differential effect of various SCFA fractions on the manifestations of cirrhosis due to fatty liver disease of various etiologies. Other limitations include the fact that all of the patients included were Caucasian and the fact that we could not control the diet of patients prior to their enrollment in the study. Further studies involving people of other races and the application of a standardized diet are required in order to verify our findings.

## 5. Conclusions

Our results complement data on changes in the intestinal microbiota in alcoholic liver cirrhosis and MAFLD-related cirrhosis. The SCFA-producing metabolic activity of the microbiota was more reduced in MAFLD cirrhosis patients than in alcoholic cirrhosis patients. Disturbances in this metabolic activity of the microbiota may be implicated in distinct pathogenetic pathways depending on the etiological factor of cirrhosis.

## Figures and Tables

**Table 1:** Main characteristics of the patients with cirrhosis and controls.

	Patients with Cirrhosis (n = 40)	Healthy Controls (n = 20)	*p*
Age, years	57 [51–64]	56 [52–59]	0.712
Male/Female	21/19	9/11	0.392
Body mass index, kg/m^2^	29.2 [24.5–32.0]	26.4 [24.6–26.8]	0.044
Waist, cm	113 [102–119]	87 [81–93]	<0.001
Serum cholesterol, mmol/L	4.5 [3.4–5.9]	4.4 [4.2–4.8]	0.975
Serum triglycerides, mmol/L	1.57 [1.21–1.72]	0.83 [0.61–1.27]	<0.001
Serum glucose, mmol/L	6.1 [5.1–7.4]	4.8 [4.6–5.0]	<0.001
Alanine aminotransferase, U/L	42 [22–78]	19 [17–24]	<0.001
Aspartate aminotransferase, U/L	50 [38–89]	16 [11–19]	<0.001
Hemoglobin, g/L	130 [120–139]	135 [132–138]	0.086
Alcohol consumption, doses/ week	27 [1–36]	1 [0–2]	0.001

**Table 2:** Main characteristics of patients with cirrhosis due to alcoholic and non-alcoholic (metabolic-associated) fatty liver disease (the ALC and MET groups, respectively).

	ALC (n = 24)	MET (n = 16)	*p*
Age, years	55 [44–62]	60 [54–65]	0.132
Male/Female	15/9	6/10	0.110
Body mass index, kg/m^2^	25.2 [22.9–29.4]	32.1 [30.7–33.1]	<0.001
Waist, cm	108 [98–114]	118 [116–124]	<0.001
Child–Pugh score	6 [5–7]	5 [5–7]	0.610
Child–Pugh class, A/B	17/7	11/5	0.580
Esophageal varices, grade 0–1/2–3	19/5	11/5	0.351
Ascites, present/absent	6/18	5/11	0.467
Minimal hepatic encephalopathy, present/absent	4/20	4/12	0.399
Serum total protein, g/L	71.0 [67.5–75.5]	70.5 [67.5–76.5]	0.978
Serum albumin, g/L	39.6 [37.2–44.7]	37.1 [34.2–40.8]	0.068
Serum total bilirubin, μmol/L	28.6 [23.8–33.4]	20.2 [13.6–27.4]	0.007
Serum cholesterol, mmol/L	4.5 [2.8–7.0]	4.4 [3.7–5.2]	0.934
Serum HDL cholesterol, mmol/L	1.4 [0.9–2.5]	1.1 [0.9–1.3]	0.113
Serum LDL cholesterol, mmol/L	2.6 [2.1–3.7]	2.7 [2.0–3.3]	0.638
Serum triglycerides, mmol/L	1.32 [1.05–1.69]	2.14 [1.65–2.65]	<0.001
Serum uric acid	353 [288–428]	247 [217–274]	<0.001
Serum glucose, mmol/L	5.8 [4.6–6.7]	6.7 [5.8–8.1]	0.028
Alanine aminotransferase, U/L	40 [18–75]	42 [31–78]	0.294
Aspartate aminotransferase, U/L	55 [34–112]	49 [43–70]	0.782
Gamma glutamyl transferase, U/L	77 [44–758]	60 [53–68]	0.464
Alkaline phosphatase, U/L	98 [87–120]	91 [70–105]	0.163
C-reactive protein, mg/L	3.6 [1.7–6.9]	2.5 [1.7–4.4]	0.214
Fibrinogen, g/L	3.2 [2.6–3.8]	4.2 [3.8–4.4]	<0.001
Prothrombin index (Quick test), %	75 [64–91]	81 [77–86]	0.269
IgG, g/L	10.9 [9.4–13.9]	15.6 [13.1–17.0]	0.011
IgM, g/L	1.6 [1.5–1.8]	1.2 [1.1–1.3]	<0.001
Hemoglobin, g/L	130 [115–139]	130 [124–138]	0.525
White blood cells, 10^9^/L	5.5 [4.8–6.1]	5.8 [4.6–6.2]	0.751
Neutrophils, 10^9^/L	2.8 [2.3–4.1]	2.9 [2.5–3.1]	0.890
Lymphocytes, 10^9^/L	1.5 [1.1–2.2]	2.1 [1.6–2.6]	<0.024
Platelets, 10^9^/L	145 [99–187]	147 [103–180]	0.761
Portal vein diameter, cm	12.5 [11.7–13.7]	12.1 [11.4–12.8]	0.258
Splenic vein diameter, cm	7.0 [6.4–8.8]	7.8 [6.6–8.5]	0.761
Alcohol consumption, doses/week	36 [32–38]	1 [0–1]	<0.001

**Table 3:** Fecal short-chain fatty acid (SCFA) levels in patients with cirrhosis due to alcoholic and non-alcoholic (metabolic-associated) fatty liver disease (the ALC and MET groups, respectively), as well as in healthy controls (the CON group).

	ALC (n = 24)	MET (n = 16)	CON (n = 20)	ALC vs. MET	ALC vs. CON	MET vs. CON
Fecal SCFA, mg/g	5.31 [3.65–7.11]	3.20 [2.13–4.22]	10.2 [9.76–10.7]	<0.001	<0.001	<0.001
Fecal acetate, mg/g	3.14 [2.30–4.49]	2.12 [1.03–2.28]	5.87 [5.65–6.04]	<0.001	<0.001	<0.001
Fecal propionate, mg/g	1.11 [0.78–1.36]	0.58 [0.46–0.81]	1.77 [1.70–1.83]	<0.001	<0.001	<0.001
Fecal butyrate, mg/g	0.68 [0.46–1.10]	0.35 [0.28–0.48]	1.69 [1.66–1.77]	0.001	<0.001	<0.001
Fecal isoacids, mg/g	0.27 [0.22–0.33]	0.23 [0.21–0.31]	0.62 [0.59–0.64]	0.276	<0.001	<0.001
Fraction of acetate, %	64.5 [60.2–66.9]	62.8 [56.3–66.8]	61.8 [56.0–67.5]	0.320	0.458	0.927
Fraction of propionate, %	21.0 [18.6–24.7]	22.7 [19.8–26.3]	19.1 [8.7–19.8]	0.923	0.017	0.043
Fraction of butyrate, %	14.8 [11.3–17.8]	12.9 [11.3–15.0]	17.1 [15.3–21.0]	0.590	0.008	0.033
Fraction of isoacids, %	5.8 [3.1–7.0]	8.2 [7.4–10.0]	5.9 [5.8–6.0]	<0.001	0.860	<0.001
Isoacid/unbrached acid ratio	0.06 [0.03–0.08]	0.08 [0.07–0.11]	0.07 [0.07–0.07]	<0.001	0.564	<0.001

**Table 4:** The fecal levels of SCFAs and their fractions in patients with and without minimal hepatic encephalopathy in cirrhosis due to alcoholic and non-alcoholic (metabolic-associated) fatty liver diseases.

Alcoholic Fatty Liver Disease			
	Minimal Hepatic Encephalopathy Present (n = 4)	Minimal Hepatic Encephalopathy Absent (n = 20)	*p*
Fecal SCFA, mg/g	4.95 [4.11–6.72]	5.56 [3.65–7.11]	0.698
Fecal acetate, mg/g	3.03 [2.30–4.28]	3.14 [2.29–4.49]	1.000
Fecal propionate, mg/g	0.95 [0.63–1.36]	1.11 [0.88–1.36]	0.588
Fecal butyrate, mg/g	0.52 [0.36–1.05]	0.76 [0.49—1.10]	0.670
Fecal isoacids, mg/g	0.28 [0.26–0.36]	0.25 [0.21–0.33]	0.373
**Non-alcoholic (metabolic-associated) fatty liver disease**			
	**Minimal hepatic encephalo-** **pathy present (n = 4)**	**Minimal hepatic encephalopathy absent (n = 12)**	*p*
Fecal SCFA, mg/g	4.22 [3.75–4.40]	3.29 [2.32–3.86]	0.034
Fecal acetate, mg/g	2.45 [2.17–2.50]	1.75 [1.26–2.28]	0.025
Fecal propionate, mg/g	0.72 [0.63–0.82]	0.60 [0.43–0.80]	0.431
Fecal butyrate, mg/g	0.48 [0.45–0.50]	0.37 [0.34–0.43]	0.060
Fecal isoacids, mg/g	0.38 [0.37–0.42]	0.27 [0.23–0.33]	0.009

## Data Availability

Data are available upon reasonable request from the corresponding author. The data are not publicly available because it is not recommended by the local ethics committee.
